# Evaluation by Flow Cytometry of Mature Monocyte Subpopulations for the Diagnosis and Follow-Up of Chronic Myelomonocytic Leukemia

**DOI:** 10.3389/fonc.2018.00109

**Published:** 2018-04-12

**Authors:** Tiphanie Picot, Carmen Mariana Aanei, Pascale Flandrin Gresta, Pauline Noyel, Sylvie Tondeur, Emmanuelle Tavernier Tardy, Denis Guyotat, Lydia Campos Catafal

**Affiliations:** ^1^Laboratoire d’Hématologie, CHU de Saint-Etienne, Saint-Etienne, France; ^2^Département d’Hématologie et Thérapie Cellulaire, Institut de Cancérologie Lucien Neuwirth, Saint-Etienne, France

**Keywords:** chronic myelomonocytic leukemia, flow cytometry, monocytic subpopulations, peripheral blood monocytosis, next-generation sequencing, karyotype

## Abstract

Chronic myelomonocytic leukemia (CMML) is a myelodysplastic/myeloproliferative neoplasm, characterized by persistent monocytosis and dysplasia in at least one myeloid cell lineage. This persistent monocytosis should be distinguished from the reactive monocytosis which is sometimes observed in a context of infections or solid tumors. In 2015, Selimoglu-Buet et al. observed an increased percentage of classical monocytes (CD14^+^/CD16^−^ >94%) in the peripheral blood (PB) of CMML patients. In this study, using multiparametric flow cytometry (MFC), we assessed the monocytic distribution in PB samples and in bone marrow aspirates from 63 patients with monocytosis or CMML suspicion, and in seven follow-up blood samples from CMML patients treated with hypomethylating agents (HMA). A control group of 12 healthy age-matched donors was evaluated in parallel in order to validate the analysis template. The CMML diagnosis was established in 15 cases in correlation with other clinical manifestations and biological tests. The MFC test for the evaluation of the repartition of monocyte subsets, as previously described by Selimoglu-Buet et al. showed a specificity of 97% in blood and 100% in marrow samples. Additional information regarding the expression of intermediate MO2 monocytes percentage improved the specificity to 100% in blood samples allowing the screening of abnormal monocytosis. The indicative thresholds of CMML monocytosis were different in PB compared to BM samples (classical monocytes >95% for PB and >93% for BM). A decrease of monocyte levels in PB and BM, along with a normalization of monocytes distribution, was observed after treatment in 4/7 CMML patients with favorable evolution. No significant changes were observed in 3/7 patients who did not respond to HMA therapy and also presented unfavorable molecular prognostic factors at diagnosis (*ASXL1, TET2*, and *IDH2* mutations). Considering its simplicity and robustness, the monocyte subsets evaluation by MFC provides relevant information for CMML diagnosis.

## Introduction

Chronic myelomonocytic leukemia (Chronic myelomonocytic leukemia) is a clonal malignant hematological disorder characterized by monocytosis and myeloid dysplastic features (1). According to the World Health Organization (WHO) classification, it has been recognized as a distinct entity from myelodysplastic syndromes and grouped with other rare myeloid malignancies that combine myeloproliferative and dysplastic traits (2). Nowadays, the biological diagnosis of CMML is based on the presence of an increased level of monocytes in peripheral blood (PB) (>10%, >1 × 10^9^/L, for at least 3 months) (3, 4). However, it may be difficult to discriminate CMML from reactive monocytosis (>1 × 10^9^/L) or prefibrotic myelofibrosis using only these criteria. For these reasons, multiparametric flow cytometry (MFC) was tested to evaluate the phenotypic profile in bone marrow (BM) or blood samples to find specific changes related to CMML. The study conducted by Shen et al. in 118 CMML patients, promotes integration of MFC data with other clinical and biological tools in the diagnosis of CMML (5). They evaluated all myeloid compartments (immature and mature) in BM aspirates and found alterations in granulocytic maturation in more than two-thirds of CMML patients (5). Likewise, they observed an increased expression of mature myelomonocytic markers on CD34^+^ myeloblasts, including CD64, CD15, and rarely, CD11b, in a small subset of CMML cases (20 of 118, 17%) (5). However, the authors observed that immunophenotypic changes in monocytes, using the criteria established by International Workshops from the European LeukemiaNet Working Group, are not specific for CMML or other MDS settings, but make the neoplastic process visible if present (5).

A startling idea suggests that monocyte subpopulations in blood are a “mirror of disrupted homeostasis and disease” (6). Therefore, it is worth evaluating for the mature monocyte compartment. Three subpopulations of mature monocytes (classical monocytes, noted hereafter as MO1; intermediate monocytes, MO2 and nonclassical, MO3) were described by the Nomenclature Committee of the International Union of Immunological Societies (NCIUIS) (7). This subdivision was validated using gene expression profiling (8–10). Recently, Selimoglu-Buet et al. demonstrated, in CMML patients, an increase in the fraction of MO1 CD14^+^/CD16^−^ monocytes (>94% for a specificity of 95.1% and a sensitivity of 90.6%) at the expense of MO2 (CD14^+^/CD16^+^) and MO3 (CD14^+low^/CD16^+^) cell fractions (11).

The aim of this study was to evaluate by MFC, the monocyte subsets, using Selimoglu-Buet test in patients showing PB monocytosis and clinical suspicion of CMML compared to “healthy” subjects and to patients carrying other hematological diseases, in order to validate this test for routine practices. The test shows excellent sensitivity and specificity for CMML diagnosis, allowing exclusion of reactive monocytosis, and permitting monitoring of CMML treatment.

## Materials and Methods

### Study Group

The study group consisted of 63 cases of monocytosis or CMML suspicion analyzed in real time, which were referred to the Institut de Cancérologie Lucien Neuwirth between March 2016 and December 2017 for diagnosis or follow-up after chemotherapy. The diagnosis of CMML was established according to the current WHO criteria (12) by a combination of clinical findings, morphologic evaluation of PB, bone marrow specimens, conventional cytogenetic and molecular analysis. The “reactive” monocytosis was considered (1) in a context of transient monocytosis (<6 months) or inflammatory disease, (2) when BM examination showed few or absent dysplastic signs, a normal karyotype and the absence of mutations by next-generation sequencing (NGS). The final distribution of the cases was CMML (*n* = 15), reactive monocytosis (*n* = 36), and hematological malignancies (*n* = 12), including chronic myeloid leukemia (*n* = 1), myelodysplastic syndromes (*n* = 2), acute myeloid leukemia (*n* = 4), multiple myeloma (*n* = 1), and myeloproliferative neoplasms (*n* = 4). In three patients with CMML and in one patient with reactive monocytosis, we evaluated both bone marrow and blood samples. In addition, monocytes subsets were evaluated in seven CMML samples (BM, *n* = 3 or PB, *n* = 4) during the clinical follow-up. Detailed characteristics of these groups are shown in Table 1. Twelve age-matched healthy subjects were analyzed in order to evaluate the robustness of the gating strategy.

**Table 1:** Patients characteristics.

	Healthy donors (*n* = 12)	Reactive monocytosis (*n* = 36)	Chronic myelomonocytic leukemia (CMML) (*n* = 15)	Non-CMML (*n* = 12)
**Samples type, *n***				
Bone marrow	1	4	10	5
Peripheral blood	11	32	5	7
Age mean, *n* (range)	64 (41–92)	67 (12–89)	80 (62–91)	71 (64–86)
**Gender, *n***				
Male	6	25	8	6
Female	6	11	7	6
Monocytes count (mean, *n* x 10^9^/L) (range)	0.55 (0.28–0.78)	2.49 (0.08–26.86)	3.88 (1.4–21.92)	2.30 (1.49–3.58)
CMML 1/CMML 2			14/1	

The “Comité de Protection des Personnes” (Independant Ethics Committee) Sud-Est 1 from University Hospital of Saint-Etienne, France has reviewed and has given ethical approval for the study. All patients gave informed consent according to the institutional procedures.

### MFC Staining and Analysis

**Figure 1:**
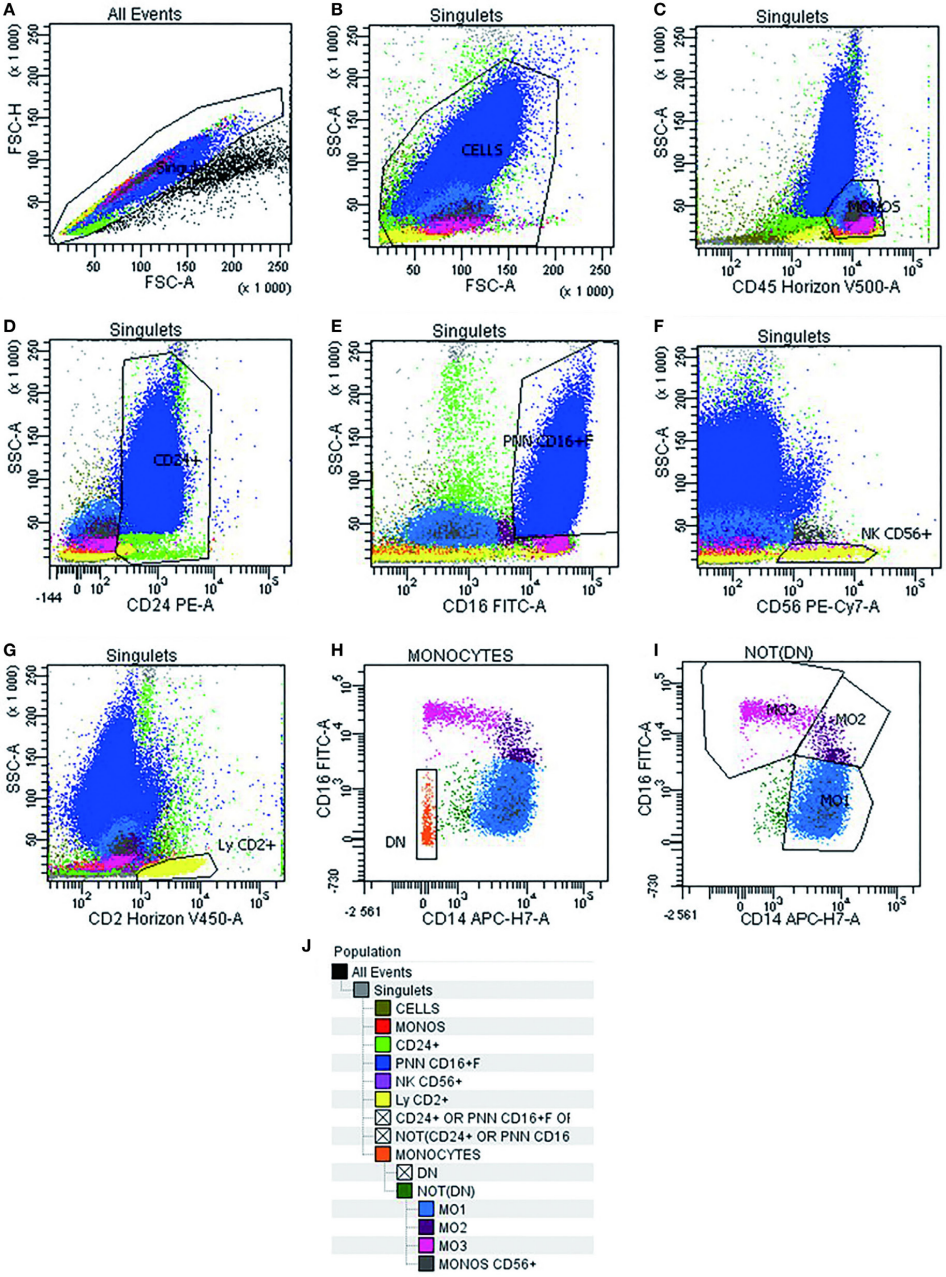
Gating strategy used in multiparametric flow cytometry analysis to detect different subsets of monocytes. Doublets exclusion on FSC-A/FSC-H dot plot **(A)**. Live cells on FSC-A/SSC-A **(B)**. Monocytes identification as CD45^+^/SSC intermediate cells **(C)**. Invert gate and exclusion of cells expressing CD24^+^, granulocytes expressing CD16^+high^, and lymphocytes expressing CD56^+^ and CD2^+^ **(D–G)**. Exclusion of residual cells CD14^−^ CD16^−^ **(H)**. Characterization of human monocyte subsets into CD14^+^/CD16^−^ (MO1, conventional), CD14^+^/CD16^+^ (MO2, intermediate), and CD14^low^/CD16^+^ (MO3, unconventional) subsets **(I)**. Cell hierarchy **(J)**.

Multiparametric flow cytometry analysis of monocyte subsets was performed on whole PB and BM samples collected on EDTA. Erythrocyte lysis was performed using FACS lysing solution (BD Biosciences, CA, USA). Cell surface staining of 10^6^ cells was performed using a combination of antibodies (Table 2) for 15 min at room temperature, sheltered from light. At least 300,000 total events and 30,000 events in the monocyte subpopulation were acquired (FACS Canto II, BD Biosciences). Data were analyzed using Diva software version 6.1.3 (BD Biosciences). Cytometer settings were established conforming to EuroFlow procedures, and the instrument quality control was checked on a daily basis (13). The monocyte subsets were identified following an exclusion gating strategy as described by Selimoglu-Buet (11). Briefly, monocytes were gated on a CD45/SSC dot plot followed by the exclusion of T lymphocytes expressing CD2, of NK lymphocytes expressing CD2, CD16, and CD56, of memory B-lymphocytes CD24^+^, of other residual contaminating cells CD14^−^ and CD16^−^, and of granulocytes expressing CD16^+high^. Thereafter, the CD16 and CD14 markers were used to discriminate the subsets of mature monocytes (MO1 CD14^+^/CD16^−^, conventional monocytes; MO2 CD14^+^/CD16^+^, intermediate monocytes; and MO3 CD14^low^/CD16^+^, unconventional monocytes) (Figure 1). The distribution of monocytes in different subsets was reported as the percentage of the total monocytes. Furthermore, we assessed monocyte distribution stability by measuring MO1, MO2, and MO3 fractions in different time points: within 24 and 48 h after harvesting (*n* = 5).

**Table 2:** List of antibodies used for multiparametric flow cytometry.

Antigen	Antibodies	Clone (isotype)	Fluorochrome	Company	Reference
CD45	Mouse anti-human CD45	HI30 (mouse IgG1, κ)	Pacific orange (V-500)	Beckton Dickinson	560777
CD24	Mouse anti-human CD24	ML5 (mouse IgG2a, κ)	PE	Beckton Dickinson	555428
CD2	Mouse anti-human CD2	TS1/8 (mouse IgG1, κ)	Pacific blue (V-450)	Biolegend	309216
CD14	Mouse anti-human CD14	MφP9 (mouse BALB/c IgG2b, κ)	APC-H7	Beckton Dickinson	641394
CD16	Mouse anti-human CD16	CLB-FcR-gran/1, 5D2 (mouse IgG2a)	FITC	PeliCluster Sanquin	M1389
CD56	Mouse anti-human CD56	N901, NKH-1 (mouse IgG1)	PE-CY7	Beckman Coulter	A21692

### Morphologic Examination

The light microscopic assessment of PB smears and BM aspirates evaluated after May–Grunwald–Giemsa staining was performed for each case by two experimented pathologists. The dysplastic changes were reported according to WHO recommendations (12).

### Next-Generation Sequencing (NGS)

Genomic DNA samples were tested in 13/15 CMML patients, by NGS, using a custom designed myeloid panel, based on an AmpliSeq strategy (Life Technologies, CA, USA). The panel addressed 24 recurrently mutated genes in myeloid malignancies: *ASXL1, CALR, CSF3R, DNMT3A, EZH2, FLT3, GATA2, IDH1, IDH2, JAK2, KIT, KRAS, NPM1, NRAS, PTPN11, RUNX1, SETBP1, SF3B1, SRSF2, TET2, TP53, U2AF1*, and *WT1*. Libraries were constructed using the Ion AmpliSeq Library Kit v2.0 according to the manufacturer’s instructions. Amplification of libraries, loading on 316 chip V2, was performed with the Ion Chief System^®^, while sequencing was executed using the Ion PGM^®^ machine (Life Technologies). Ion Reporter^®^ and NextGENe^®^ v.2.3.4 (SoftGenetics, USA) software were used to perform bioinformatics analysis, including optimized signal processing, base calling, sequence alignment (hg19 reference), and variant analysis. Variants detected with a frequency of 2% or higher on both strands were considered as present. Sanger sequencing was performed for all patients to identify the ASXL1 c.1934dupG (p.G5646Wfs*12), as this mutation, occurring in a homopolymer region, is not detected with our NGS approach. Furthermore, all variants not referenced as mutational hotspots in international databases (Ensembl, Cosmic, IARC TP53), and detected with a VAF >20%, were confirmed by Sanger sequencing.

### Karyotype

Bone marrow samples for cytogenetic analysis were obtained for 13/15 CMML patients at the time of diagnosis. Karyotypes were analyzed after 24-h culture following standard procedures. The chromosomes were stained by R- and G-banding. At least 20 metaphases were analyzed. Results were interpreted and reported according to the International System for Human Cytogenetic Nomenclature (ISCN, 2013 and 2016) (14).

### Statistical Analysis

The nonparametric Fisher’s test was used to compare distributions between groups. Cut-offs were estimated in the learning cohort by maximizing the Youden index (J = sensitivity + specificity − 1). These cut-offs were compared to the classical CD14^+^/CD16^−^ monocyte count cut-off of 1 × 10^9^/L.

Analysis and figure plotting were performed with GraphPad Prism 5 software (GraphPad, CA, USA).

## Results

### Distribution of Monocyte Subsets

**Figure 2:**
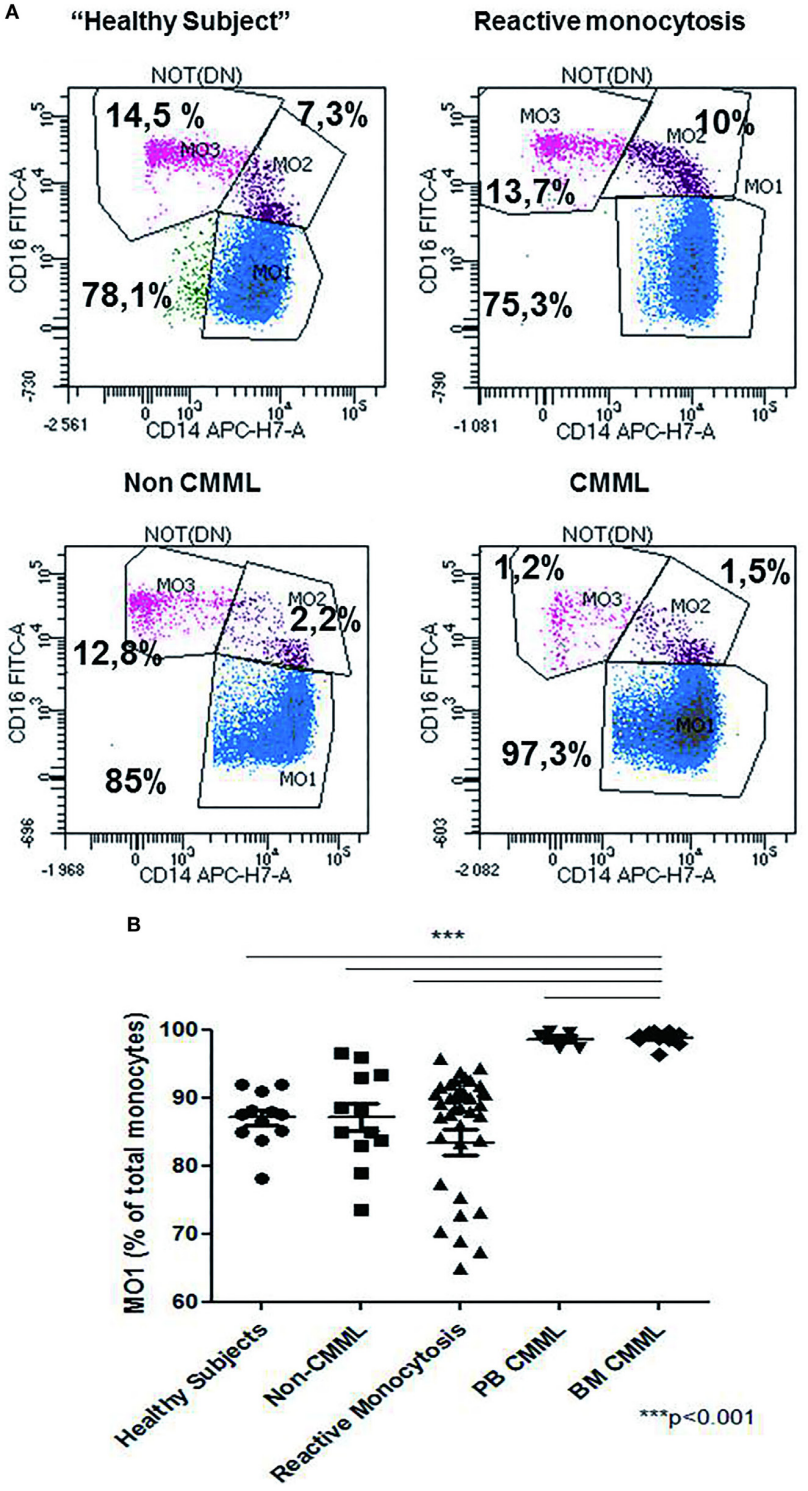
Repartition of monocytes subsets in different groups of cases. Monocyte subset repartition in distinct groups [*n* = 12 “healthy subjects,” *n* = 15 chronic myelomonocytic leukemia (CMML), *n* = 36 reactive monocytosis, *n* = 12 hematological diseases or non-chronic myeloid leukemia (CMML) (*n* = 1), MDS (*n* = 2), AML (*n* = 4), myeloma (*n* = 1), and myeloproliferative syndromes (*n* = 4)]. For each group, the monocytes subsets expressed as percentage of total monocytes are displayed **(A)**. Relative frequencies of the MO1 subset in different groups (*n* = 12 “healthy subjects,” *n* = 12 non-CMML, *n* = 36 reactive monocytosis >1 × 10^9^, *n* = 5 peripheral blood CMML, and *n* = 10 bone marrow CMML) **(B)**. Fisher test was performed in order to compare each group of cases with healthy subjects. ***p < 0.001.

The relative frequencies of monocyte subsets in the “healthy” group were 87.04% ± 3.7 MO1, 4.2% ± 1.79 MO2, and 8.7% ± 3.98 MO3 and in patients with reactive monocytosis 82.7% ± 13.61 MO1, 8.4% ± 11.97 MO2, and 8.6 ± 6.41 MO3 (Figure 2A). CMML cases demonstrated a significant increase in MO1 percentage (*p* < 0.001) compared to MO2 and MO3 subsets in PB (97.2% ± 4.0 MO1, 1.8% ± 1.8 MO2, and 0.9% ± 2.3 MO3) and in BM (98.7% ± 3.5 MO1, 0.6% ± 0.9 MO2, and 0.5% ± 2.9 MO3) (Figure 2A). In CMML patients, dysplastic signs of one or more BM myeloid cells were observed, along with the presence of clonal genetic abnormalities (the most frequent mutations were *ASXL1*: *n* = 6/13 patients, *SRSF2*: *n* = 7/13 patients, and *TET2*: *n* = 9/13 patients) (Table 3). 4/13 CMML patients harbored an abnormal karyotype: three patients presented recurrent alterations found in myeloid malignancies (monosomy 7, trisomy 8, and trisomy 14), whereas the fourth one presented genetic abnormalities which were non-recurrent in myeloid diseases.

**Table 3:** Mutational profile of chronic myelomonocytic leukemia patients.

	1	2	3	4	5	6	7	8	9	10	11	12	13
% MO1	99.60%	99.10%	98.60%	99.50%	97.40%	96.30%	98.60%	98.00%	99.10%	99.30%	99.70%	99.70%	97.30%
*TET2*													
*ASXL1*													
*SRSF2*													
*IDH2*													
*SF3B1*													
*SETBP1*													
*CSF3R*													
*NRAS*													
*NPM1*													
*PTPN11*													
*DNMT3A*													
*JAK2*													
*FLT3*													
*KIT*													
*RUNX1*													
*WT1*													
*U2AF1*													
*EZH2*													
*IDH1*													
*KRAS*													
*CALR*													
*TP53*													
Trisomy 14													
Monosomy 7													
Trisomy 8													
Other genetic abnormalities non-recurrent in myeloid diseases													
Normal karyotype													

*Red color shows the presence of molecular abnormalities and violet color shows the presence of chromosomal abnormalities in each patients*.

Patients carrying other myeloid malignancies showed a similar pattern of monocyte distribution to those having reactive monocytosis (87.1% ± 7.07 MO1, 5.4% ± 5.79 MO2, and 7% ± 4.65 MO3) (Figure 2B).

**Figure 3:**
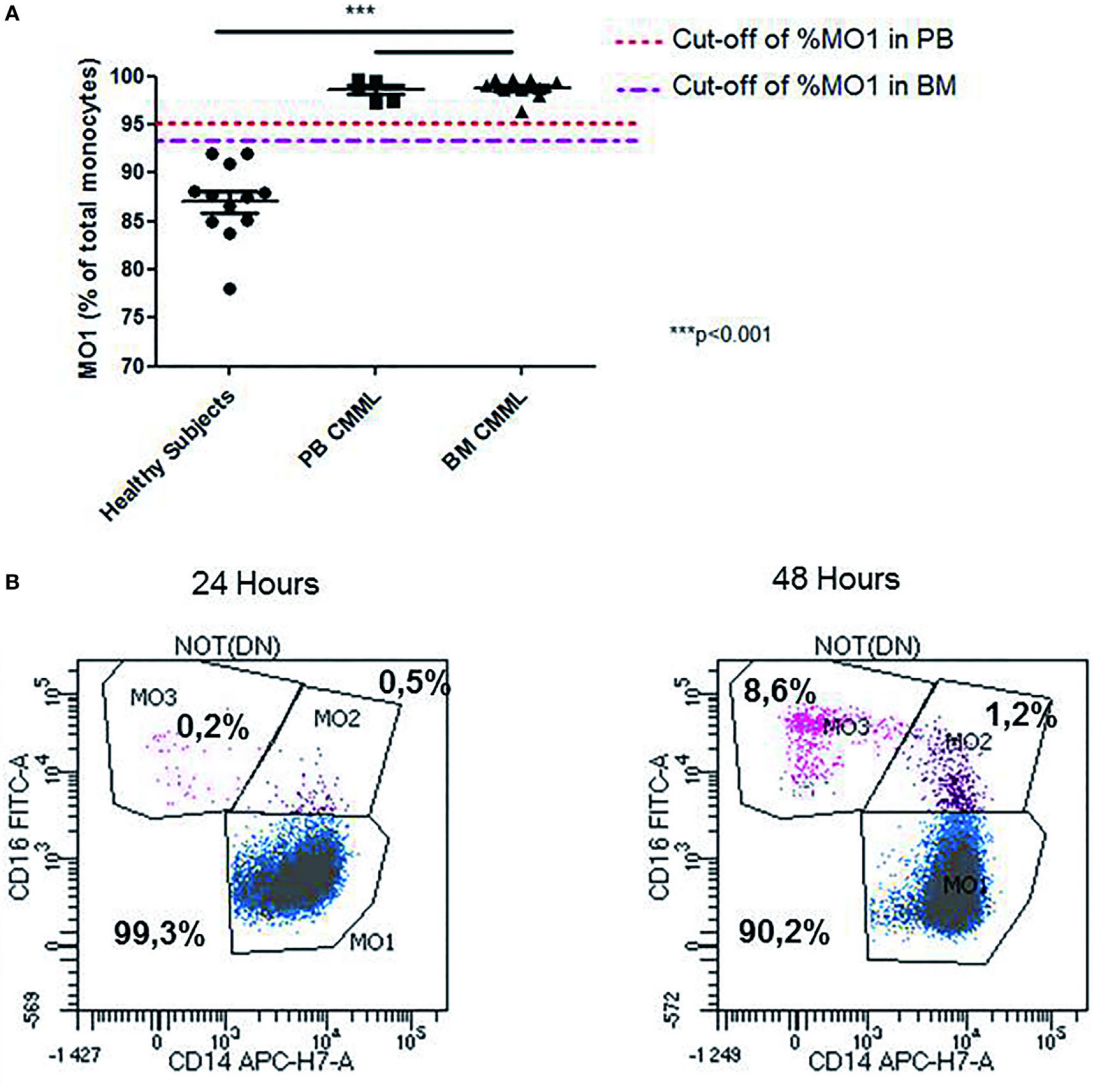
Abnormal repartition of monocyte subsets in chronic myelomonocytic leukemia (CMML). Evaluation of the MO1 percentage cut-off subset in peripheral blood (PB) (*n* = 5) and bone marrow (BM) (*n* = 10) in the CMML group **(A)**. The MO1 percentage cut-off points were compared with the threshold set (1 × 10^9^/L) for the PB monocytosis. The differences between the values obtained in the MO1 percentage in BM and PB for the CMML group compared with those detected in the healthy subjects were statistically significant (***p < 0.001; Fisher test). Representative example for the differences observed in the distribution of monocytes subset when the test was performed at 24 and 48 h after sample harvesting. The monocytes subsets are expressed as a percentage of total monocytes **(B)**.

Interestingly, we observed changes in MO1, MO2, and MO3 distribution in two CMML samples when MFC was performed at 24 and 48 h after harvesting. While MO1 percentage was consistent with CMML at 24 h (>96%), the analysis at 48 h showed a monocyte profile that was similar to that observed in reactive monocytosis (Figure 3B).

### The Contribution of MFC in Detection of CMML Monocytosis

The statistical analysis showed that the cut-off percentage of MO1 suggestive of CMML in PB was 95% (97% specificity, 100% sensitivity, *p* < 10^−4^, χ^2^ test, Youden index = 0.97) and 93% in BM (100% specificity, 100% sensitivity, *p* < 10^−4^, χ^2^ test, Youden index = 1) (Figure 3A). Using these cut-offs, the MFC screening test was consistent with CMML diagnosis in 100% (15/15) of the cases evaluated in BM samples. The MFC results were in concordance with BM cytology, karyotype, and molecular biology. In 100% of CMML cases, the MO2 percentage was <2%, whereas in reactive monocytosis cases it was always >2% (*p* < 0.001). Four reactive monocytosis cases presented high percentage of blood MO1 monocytes (>95%), but with MO2 percentage >2%. In addition, the monocyte subpopulations screening by MFC was performed in two AML NPM1^+^ patients in remission, showing a persistent monocytosis and dysplastic signs in BM evaluation, after consolidation therapy in one case, and after allogeneic stem cell transplantation for the second case. In both cases, an increased level of MO1 monocytes (>96%) was detected by MFC. NGS revealed, in these cases, respectively, a *TET2* and an *IDH2* mutation.

### Monocyte Subset Profile During Follow-Up

**Figure 4:**
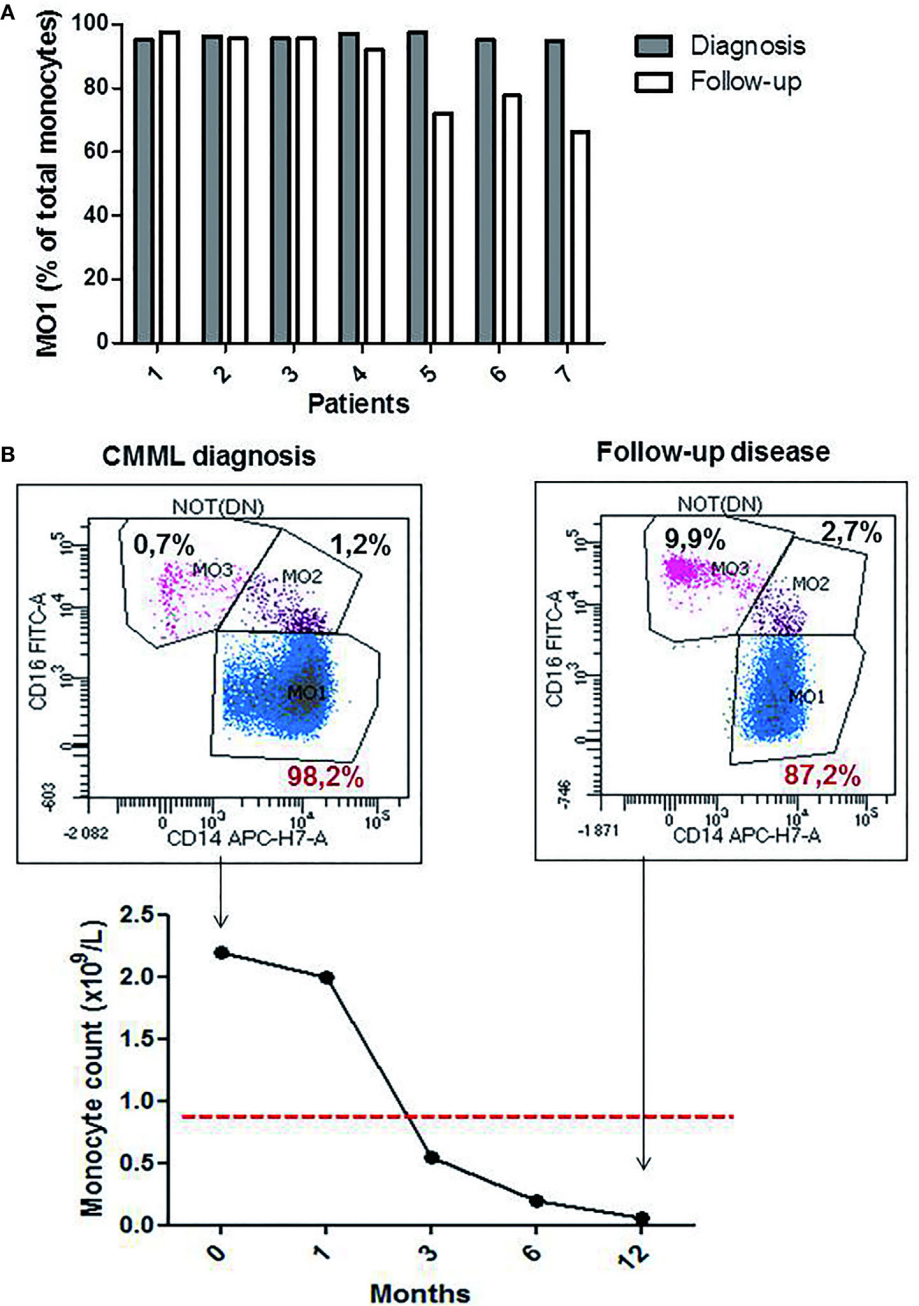
The profile of the monocytes subsets during chronic myelomonocytic leukemia (CMML) follow-up. Relative frequencies of the MO1 subsets at the diagnosis point (dark bar) versus follow-up (white bar) (*n* = 7 CMML patients). MO1 are expressed as a percentage of total monocytes **(A)**. Representative example for the evolution of % MO1 classical monocytes during the follow-up in a CMML case with good response to the hypomethylating agents therapy **(B)**.

The monocytes profile evaluation was performed in seven CMML cases during hypomethylating agents (HMA) treatment (4 PB and 3 BM samples). A significant decrease in the percentage of MO1 monocytes was observed in four patients (patients 4, 5, 6, and 7; Figure 4A) (mean of the group 77.05% ± 0.14 for MO1, 10% ± 0.03 for MO2, and 12.20% ± 0.12 for MO3) along with a return to normal values of monocyte counts (<1 × 10^9^/L). Figure 4B shows a representative example of a good response to HMA therapy. A persistence of CMML monocytosis was observed in three patients (patients 1, 2, and 3) along with an increased percentage in MO1 >95% (mean of 96.3% ± 0.02 for MO1, 2.5% ± 0.006 for MO2, and 2.3% ± 0.01 for MO3) (Figure 4A). The MFC results were confirmed in patients 1–3 by cytological analysis, and persistence of dysplastic signs in BM myeloid cells was reported.

## Discussion

With overlapping MDS and MPN features, CMML displays a complex biological and clinical heterogeneity, which is not reflected by the mutational landscape (15). This is illustrated by the absence of disease-relevant mutations that can recapitulate the CMML phenotype in mouse models (15).

The exhaustive studies evaluating BM myeloblasts, monocytes, and granulocytes showed the existence of multiple MFC abnormalities in myeloid compartments in CMML, which are closer to MDS than MPN, but also unspecific for CMML (5, 16). However, evaluation by MFC of all myeloid compartments in BM is a complex, expensive, time-consuming procedure. Therefore, MFC testing of PB monocyte subsets using a limited number of antibodies in one single tube is a promising tool for the differential diagnosis between CMML and reactive monocytosis (11). We confirm that this method is rapid and efficient in PB (NPV = 1, PPV = 0.8) and in BM (NPV = 1, PPV = 1). Significant thresholds for MO1 monocytes in CMML were observed in PB (>95%) and BM (>93%) was not previously described. The addition of MO2 evaluation to that of MO1 allowed us to reach the same levels of sensitivity and specificity in PB as in BM, as four cases with a percentage of MO1 >95% were reassigned to a diagnosis of reactive monocytosis.

The major drawback of this test is the choice of thresholds between MO1, MO2, and MO3 subpopulations. The previously published studies used an automated clustering algorithm called spanning-tree progression analysis of density-normalized events (SPADE) to confirm the gating strategy (Cytobank software) in order to avoid gaiting errors (11, 17). Therefore, in order to improve our gating strategy, an evaluation of some cases using the Infinicyt software (version 1.8) was carried out. Although, for healthy controls and reactive monocytosis the automatic population separator analysis helped for better identification of the MO1, MO2, and MO3. However, this method was not efficient in CMML cases (data not shown). We also studied the stability of monocyte subset distribution during the sample storage. We showed that the delay between sampling and testing should not exceed 24 h in order to avoid the decrease in MO1 and the increase in MO2 subsets. This pre-analytical issue has not been described in previous studies, but can explain false negative results and a lack of specificity.

A return to normal values of monocytic subtypes could be used as a simple marker in the disease follow-up. Our preliminary data revealed a normal pattern of monocytes distribution in CMML patients in remission, but further studies are needed to evaluate the usefulness of this test for disease monitoring. Three patients who showed persistent abnormal monocyte distribution had unfavorable molecular prognostic factors (i.e., *ASXL1, TET2, SRSF2*, and *IDH2* mutations) which correlated with poor response to HMA therapy.

Furthermore, this test was performed in two AML patients in remission, who exhibited constant monocytosis during treatment. MFC testing revealed an increase in MO1 subpopulation >96% which was in line with NGS findings showing a *TET2* and an *IDH2* mutation, respectively. Mutations in epigenetic modifying enzymes, such as TET2 and IDH2, are highly prevalent in CMML (18–20) and associated with DNA hypermethylation (21, 22). The diagnosis of secondary CMML was set in these cases by corroborating evidence with other biological tests and clinical manifestations.

In conclusion, using only blood monocytosis criteria, the diagnosis of CMML is difficult when dysplasia is not evident. BM cell karyotype may comfort CMML diagnosis, but anomalies are observed only in a minority of cases (less than 30%) and are not specific to this disease. Our findings suggest a hierarchy of biological tests for CMML diagnosis, first relying on the MFC test of blood samples to exclude non-CMML patients from further invasive procedures. The NGS test is in search of recurrently mutated genes in myeloid malignancies and a complete evaluation including BM exploration could be reserved for patients with abnormal MFC results.

## Ethics Statement

All patients gave informed consent according to the institutional procedures.

## Author Contributions

TP performed the experiments. TP, CA, PF-G analyzed data, wrote the article, created the tables, figures, and gave final approval. PN, ST contributed to data analysis and reviewed the article. ET, DG, LC reviewed the article and gave final approval.

## Conflict of Interest Statement

The authors declare that the research was conducted in the absence of any commercial or financial relationships that could be construed as a potential conflict of interest.

## References

[1] Bacher U Haferlach T Schnittger S Kreipe H Kröger N Recent advances in diagnosis, molecular pathology and therapy of chronic myelomonocytic leukemia. Br J Haematol (2011) 153:149–67. 10.1111/j.1365-2141.2011.08631.x 21401573

[2] Orazi A Bennett JM Germing U Brunning RD Bain BJ Thiele J Chronic myelomonocytic leukaemia. In: Swerdlow SH Campo E Harris NL Jaffe ES Pileri SA Stein H, editors. World Health Organization Classification of Tumours of Haematopoietic and Lymphoid Tissues. Lyon: IARC Press (2008). p. 76–9.

[3] Itzykson R Solary E. An evolutionary perspective on chronic myelomonocytic leukemia. Leukemia (2013) 27:1441–50. 10.1038/leu.2013.100 23558522

[4] Vardiman JW Thiele J Arber DA Brunning RD Borowitz MJ Porwit A The 2008 revision of the World Health Organization (WHO) classification of myeloid neoplasms and acute leukemia: rationale and important changes. Blood (2009) 114:937–51. 10.1182/blood-2009-03-209262 19357394

[5] Shen Q Ouyang J Tang G Jabbour EJ Garcia-Manero G Routbort M Flow cytometry immunophenotypic findings in chronic myelomonocytic leukemia and its utility in monitoring treatment response. Eur J Haematol (2015) 95(2):168–76. 10.1111/ejh.12477 25354960

[6] Van DJJM Orfao MCEVJA *Generic Methods and Means for Monitoring Disruption of Tissue Homeostasis in the Total Body* Google Patents US 2014/0024019 A1 (2014). Available from: https://patents.google.com/patent/US20140024019A1/en (Accessed: January 23, 2014).

[7] Ziegler-Heitbrock L Ancuta P Crowe S Dalod M Grau V Hart DN Nomenclature of monocytes and dendritic cells in blood. Blood (2010) 116:e74–80. 10.1182/blood-2010-02-258558 20628149

[8] Cros J Cagnard N Woollard K Patey N Zhang SY Senechal B Human CD14dim monocytes patrol and sense nucleic acids and viruses via TLR7 and TLR8 receptors. Immunity (2010) 33:375–86. 10.1016/j.immuni.2010.08.012 20832340 PMC3063338

[9] Wong KL Tai JJ-Y Wong W-C Han H Sem X Yeap WH Gene expression profiling reveals the defining features of the classical, intermediate, and nonclassical human monocyte subsets. Blood (2011) 118:e16–31. 10.1182/blood-2010-12-326355 21653326

[10] Zawada AM Rogacev KS Rotter B Winter P Marell RR Fliser D SuperSAGE evidence for CD14+CD16+ monocytes as a third monocyte subset. Blood (2011) 118:e50–61. 10.1182/blood-2011-01-326827 21803849

[11] Selimoglu-Buet D Wagner-Ballon O Saada V Bardet V Itzykson R Bencheickh L Characteristic repartition of monocyte subsets as a diagnostic signature of chronic myelomonocytic leukemia. Blood (2015) 125:3618–26. 10.1182/blood-2015-01-620781 PMC4497970 25852055

[12] Arber DA Orazi A Hasserjian R Thiele J Borowitz MJ Le Beau MM The 2016 revision to the World Health Organization classification of myeloid neoplasms and acute leukemia. Blood (2016) 127:2391–405. 10.1182/blood-2016-03-643544 27069254

[13] Kalina T Flores-Montero J van der Velden VHJ Martin-Ayuso M Böttcher S Ritgen M EuroFlow standardization of flow cytometer instrument settings and immunophenotyping protocols. Leukemia (2012) 26:1986–2010. 10.1038/leu.2012.122 22948490 PMC3437409

[14] Shaffer LG McGowan-Jordan J Schmid M An International System for Human Cytogenetic Nomenclature (ISCN 2013) Karger (2013).

[15] Ball M List AF Padron E. When clinical heterogeneity exceeds genetic heterogeneity: thinking outside the genomic box in chronic myelomonocytic leukemia. Blood (2016) 128:2381–7. 10.1182/blood-2016-07-692988 27707735

[16] Subira D Font P Villalon L Serrano C Askari E Gongora E Immunophenotype in chronic myelomonocytic leukemia: is it closer to myelodysplastic syndromes or to myeloproliferative disorders? Transl Res (2008) 151:240–5. 10.1016/j.trsl.2008.03.001 18433705

[17] Mukherjee R Barman PK Thatoi KP Tripathy R Das BK Ravindran B. Non-classical monocytes display inflammatory features: validation in sepsis and systemic lupus erythematous. Nature (2015) 5:13886. 10.1038/srep13886 26358827 PMC4566081

[18] DiNardo CD Jabbour E Ravandi F Takahashi K Daver N Routbort M IDH1 and IDH2 mutations in myelodysplastic syndromes and role in disease progression. Leukemia (2015) 30:980–4. 10.1038/leu.2015.211 26228814 PMC4733599

[19] Itzykson R Kosmider O Renneville A Gelsi-Boyer V Meggendorfer M Morabito M Prognostic score including gene mutations in chronic myelomonocytic leukemia. J Clin Oncol (2013) 31:2428–36. 10.1200/JCO.2012.47.3314 23690417

[20] Malcovati L Papaemmanuil E Ambaglio I Elena C Galli A Della Porta MG Driver somatic mutations identify distinct disease entities within myeloid neoplasms with myelodysplasia. Blood (2014) 124:1513–21. 10.1182/blood-2014-03-560227 24970933 PMC4148773

[21] Figueroa ME Skrabanek L Li Y Jiemjit A Fandy TE Paietta E MDS and secondary AML display unique patterns and abundance of aberrant DNA methylation. Blood (2009) 114:3448–58. 10.1182/blood-2009-01-200519 19652201 PMC2765680

[22] Meldi K Quin T Buchi F Droin N Sotzen J Micol JB Specific molecular signatures predict decitabine response in chronic myelomonocytic leukemia. J Clin Invest (2015) 125(5):1857–72. 10.1172/JCI78752 25822018 PMC4611703

